# Development and optimization of a microbial co-culture system for heterologous indigo biosynthesis

**DOI:** 10.1186/s12934-021-01636-w

**Published:** 2021-08-04

**Authors:** Tingting Chen, Xiaonan Wang, Lei Zhuang, Alan Shao, Yinghua Lu, Haoran Zhang

**Affiliations:** 1 Department of Chemical and Biochemical Engineering, College of Chemistry and Chemical Engineering, Xiamen University, Xiamen, 361005 Fujian China; 2 Department of Chemical and Biochemical Engineering, Rutgers, The State University of New Jersey, Piscataway, NJ 08854 USA; 3 School of Arts and Sciences, Rutgers, The State University of New Jersey, 77 Hamilton Street, New Brunswick, NJ 08901 USA; 4 The Key Lab for Synthetic Biotechnology of Xiamen City, Xiamen University, Xiamen, 361005 Fujian China; 5 Fujian Collaborative Innovation Center for Exploitation and Utilization of Marine Biological Resources, Xiamen University, Xiamen, 361005 Fujian China

**Keywords:** Indigo, *E. coli*, Modular co-culture engineering, Biosensor, Global transcription machinery engineering

## Abstract

**Background:**

Indigo is a color molecule with a long history of being used as a textile dye. The conventional production methods are facing increasing economy, sustainability and environmental challenges. Therefore, developing a green synthesis method converting renewable feedstocks to indigo using engineered microbes is of great research and application interest. However, the efficiency of the indigo microbial biosynthesis is still low and needs to be improved by proper metabolic engineering strategies.

**Results:**

In the present study, we adopted several metabolic engineering strategies to establish an efficient microbial biosynthesis system for converting renewable carbon substrates to indigo. First, a microbial co-culture was developed using two individually engineered *E. coli* strains to accommodate the indigo biosynthesis pathway, and the balancing of the overall pathway was achieved by manipulating the ratio of co-culture strains harboring different pathway modules. Through carbon source optimization and application of biosensor-assisted cell selection circuit, the indigo production was improved significantly. In addition, the global transcription machinery engineering (gTME) approach was utilized to establish a high-performance co-culture variant to further enhance the indigo production. Through the step-wise modification of the established system, the indigo bioproduction reached 104.3 mg/L, which was 11.4-fold higher than the parental indigo producing strain.

**Conclusion:**

This work combines modular co-culture engineering, biosensing, and gTME for addressing the challenges of the indigo biosynthesis, which has not been explored before. The findings of this study confirm the effectiveness of the developed approach and offer a new perspective for efficient indigo bioproduction. More broadly, this innovative approach has the potential for wider application in future studies of other valuable biochemicals’ biosynthesis.

**Supplementary Information:**

The online version contains supplementary material available at 10.1186/s12934-021-01636-w.

## Introduction

Indigo is an aromatic molecule with deep blue color and has been historically used as a natural dye. Most of the indigo used in industry is currently obtained by either plant extraction or chemical synthetic process [1]. However, these methods need to use massive amount of chemicals as extractant, reactant or catalyst, leading to a series of process economic, sustainability, and environmental problems. To address this challenge, the indigo biosynthesis using renewable materials has been explored as a robust alternative.

**Fig. 1:**
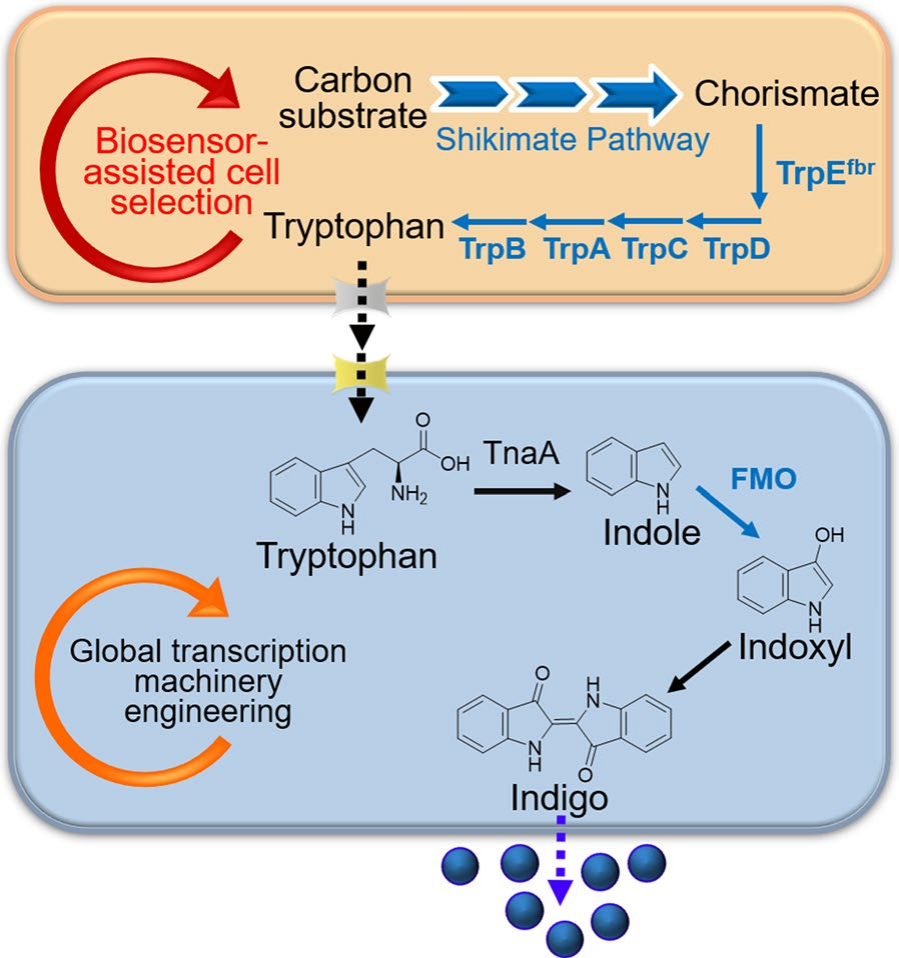
Design of *E. coli–E. coli* co-culture system for indigo biosynthesis. The pathway intermediate tryptophan was provided by the upstream strain, and the conversion of tryptophan to indigo was performed by downstream strain. Biosensor-assisted selection circuit was introduced to the upstream strain for enhancing the tryptophan provision. gTME selection was introduced into the downstream strain for improving the indigo production. Blue arrows represent the over-expressed genes

The indigo biosynthesis pathway is illustrated in Fig. 1. A carbon substrate (e.g. glucose or glycerol) is first converted to amino acid tryptophan through the central metabolism and the tryptophan pathway. Tryptophan is then further converted to indole by tryptophanase (TnaA) encoded by the *tnaA* gene. In the presence of a heterologous flavin-containing monooxygenase (FMO), indoxyl and 2-hydroxyindole are produced from indole. Spontaneous dimerization of indoxyl and 2-hydroxyindole results in the formation of indigo and indirubin, respectively [2, 3]. A few microorganisms, including *Acinetobacter sp.*, *Comamonas sp.*, and *Rhodococcus opacus* B-4, have been utilized to produce indigo in vivo [4–6]. Heterologous biosynthesis of indigo from the precursor indole or tryptophan has also been achieved in *E. coli* by utilizing phenol hydroxylase [7], naphthalene dioxygenase [8, 9], or FMO [10–12]. In addition, several studies have reported the de novo indigo bioproduction using recombinant *E. coli* strains carrying the complete pathway genes leading from glucose to indigo [8, 11, 13]. However, engineering a microbial mono-culture for the indigo biosynthesis often requires extensive genetic and metabolic engineering efforts and is restricted by the metabolic capacity of the recruited microbial hosts to accommodate the need of the biosynthesis pathway.

Recent development of microbial co-culture engineering offers a new perspective and novel opportunities for conducting microbial bioproduction. In particular, the biosynthetic pathway can be divided into separate modules, each of which can be engineered independently. A wealth of co-culture systems has been successfully developed for biosynthesis of various value-added compounds [14–17]. The co-culture engineering approach has several important advantages, such as flexibility for balancing pathway, reduction of metabolic burden, and decrease of undesired interference between pathway modules. Meanwhile, compared with the conventional mono-culture-based approach, co-culture engineering has been proven to be a better platform for adoption of novel engineering strategies. For example, it has been reported that the integration of biosensor-assisted cell selection systems into microbial co-cultures is more effective than into mono-cultures for enhancing the provision of the biosynthesis pathway intermediates and the overall bioproduction performance [16, 18, 19].

On the other hand, global transcription machinery engineering (gTME) is a powerful engineering approach that tunes the global metabolic network to fit the need of microbial biosynthesis [20]. By engineering transcription factors such as an RNA polymerase subunit, gTME perturbates the global transcriptome of the microbial host, which promotes phenotypic changes of biosynthesis performance. Although there have been many reports using gTME in microbial biosynthesis applications, they focused on engineering the mono-culture of one single microbial host. To our knowledge, there have been no previous efforts utilizing gTME in the context of microbial co-culture. Given the success of co-culture engineering and gTME in the past, their combined use in microbial biosynthesis is an interesting research area that should be explored.

In the present study, the indigo biosynthesis was established in an *E. coli–E. coli* co-culture, which was subsequently integrated with biosensor-assisted cell selection for improving the biosynthesis. Moreover, gTME approach was utilized to develop a co-culture variant with enhanced biosynthesis capability.

## Results

### Developing an *E. coli–E. coli* co-culture for the indigo biosynthesis on glucose and glycerol

The indigo biosynthetic pathway consists of tryptophan provision and its conversion to indigo (Fig. 1). To establish an *E. coli–E. coli* co-culture, the upstream tryptophan provision module and the downstream tryptophan conversion module were introduced into two *E. coli* strains, respectively. A previously constructed tryptophan over-producing strain BTP1 was employed as the upstream co-culture strain [21]. The downstream strain BD was constructed by over-expressing *Methylophaga aminisulfidivorans* flavin-containing monooxygenase (FMO) [11] in a previously engineered *E. coli* strain BH2 [22]. The co-cultivation of these two strains in one culture presented the complete biosynthetic system converting the carbon substrate to the product indigo. Previous studies [11, 23] and our own experimental results (Additional file 1: Fig. S1) demonstrated that 30 °C is the optimal temperature for the indigo synthesis by FMO. Thus, 30 °C was adopted for all the following experiments.

**Fig. 2:**
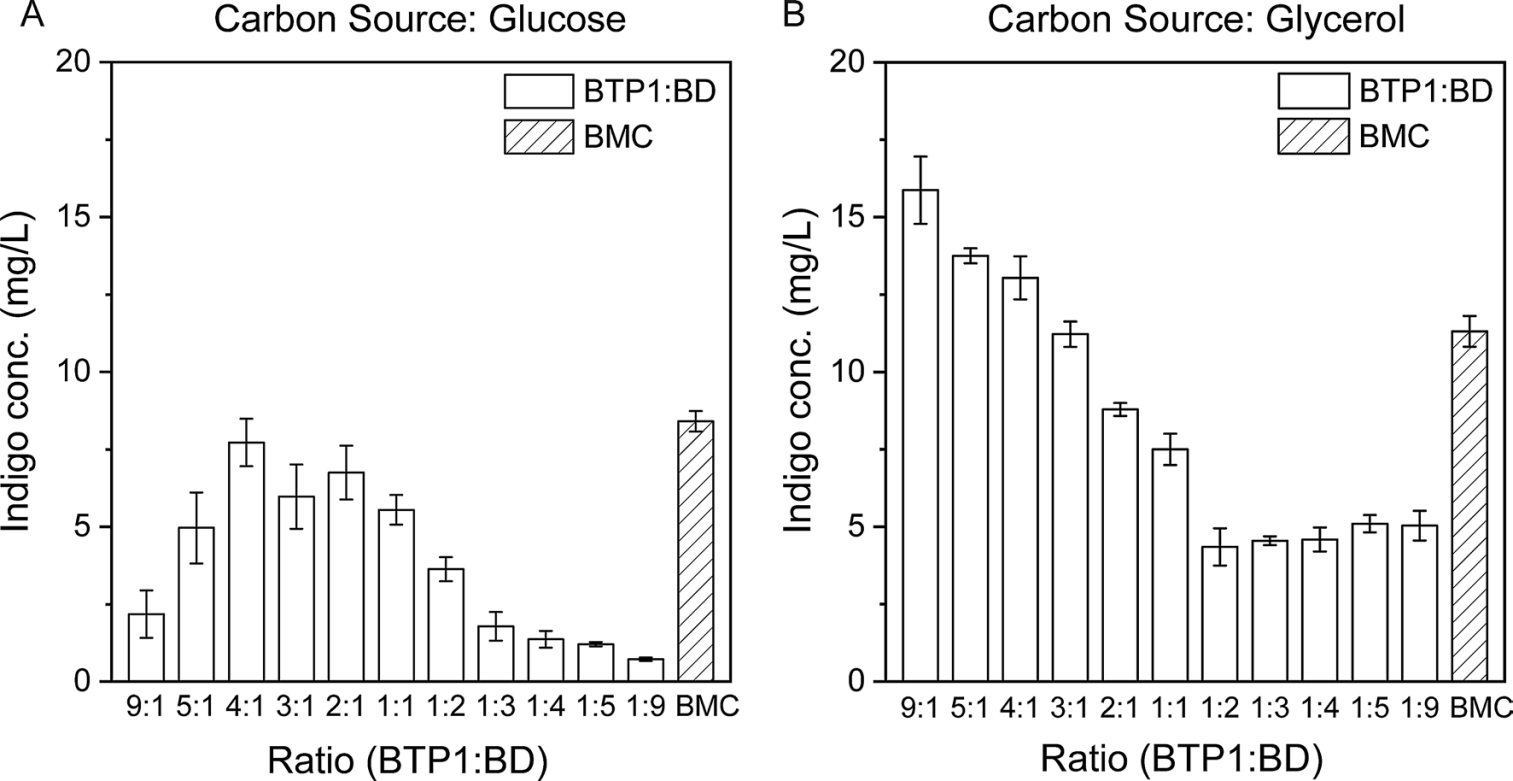
Comparison of the indigo bioproduction by the BMC mono-culture and BTP1:BD co-culture inoculated at different ratios using **A** glucose and **B** glycerol as the carbon substrate. The error bars represent the standard error of at least three biological replicates

On the other hand, two renewable carbon substrates, glucose and glycerol, were compared for their effect on the indigo biosynthesis. Glucose is one of the most commonly used sugars for microbial biosynthesis, due to its high abundance and efficient utilization by many microbes. Glycerol is a major byproduct of the biodiesel industry and has been widely used for biosynthesis of a variety of biochemical products in recent years [24–26]. As shown in Fig. 2A, when glucose was employed as the carbon substrate, the BTP1:BD co-culture showed varied indigo production performance at different inoculation ratios. At the optimal ratio of 4:1, 7.7 mg/L indigo was produced after 48 h cultivation. For comparison, the FMO gene was over-expressed in BTP1 to generate strain BMC which served as a mono-culture control strain for the de novo bioproduction of indigo. Under the same cultivation conditions, 8.4 mg/L indigo was produced by BMC.

The indigo production using glycerol as the carbon source is shown in Fig. 2B. At all inoculation ratios, the indigo concentration was higher than when glucose was used. The highest production of 15.9 mg/L was achieved at the inoculation ratio of 9:1, which was 107% higher than the optimum production on glucose. The biosynthesis improvement hereby indicated that glycerol is a better carbon source for this co-culture system. In the meantime, the monoculture control (BMC) produced 11.3 mg/L indigo from 5 g/L glycerol, 41% lower than the production by the BTP1: BD co-culture system.

### Utilizing biosensor-assisted cell selection systems to improve the indigo production

Biosensor-assisted cell selection has been proven to be an effective approach to improve the quality of the microbial population and enhance the microbial biosynthesis performance [16, 18, 21, 27–29]. For this study, this approach was adopted to engineer the upstream co-culture strain. Specifically, the growth of the engineered strain was directed by a growth-regulating gene whose expression was under the control of a tryptophan-responsive biosensor: low tryptophan-producing cells were inhibited for growth, whereas the growth of high tryptophan producing cells were promoted. Two tryptophan-responsive biosensor-assisted cell selection systems, *tnaC*-*tetA* and TrpR-P*mtr*-*hipA*, were introduced into the upstream strain (Additional file 1: Fig. S2). For the *tnaC-tetA* system, the leader peptide gene *tnaC* was used to control the tetracycline resistance gene *tetA* [16]. For the TrpR-P*mtr*-*hipA* system, P*mtr* (promoter of *E. coli* gene *mtr*), was used to control the expression of a toxin gene *hipA* [21]. The resulting strains BTS (*tnaC-tetA*) and BTS1 (TrpR-P*mtr*-*hipA*) containing these two systems have been previously characterized [16, 21], and for this work, they were co-cultivated with the downstream strain BD for the indigo biosynthesis, respectively.

**Fig. 3:**
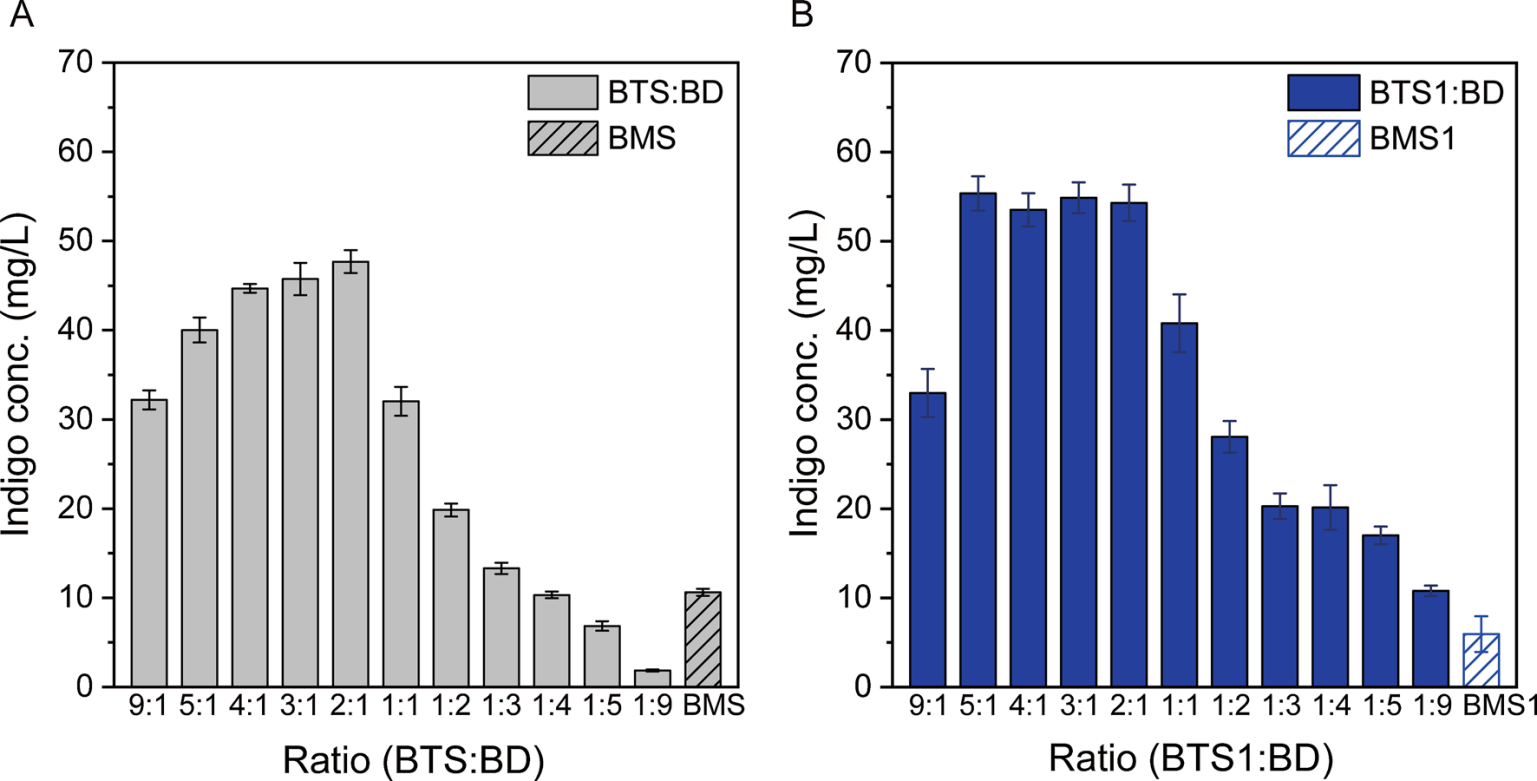
Comparison of the indigo bioproduction on glycerol using different biosensor-assisted cell selection systems. **A** The indigo production by the BTS:BD co-culture and the BMS mono-culture containing the *tnaC*-*tetA* system. **B** The indigo production by the BTS1:BD co-culture and the BMS1 mono-culture containing the P*mtr*-*hipA* system. The error bars represent the standard error of at least three biological replicates

As shown in Fig. 3, the indigo production on glycerol were significantly improved when *tnaC-tetA* (Fig. 3A) or TrpR-P*mtr*-*hipA* (Fig. 3B) were recruited. In the BTS:BD system (containing *tnaC-tetA*), the highest indigo production was 47.7 mg/L at the inoculation ratio of 2:1. The highest indigo production by the BTS1:BD co-culture system (containing TrpR-P*mtr*-*hipA*) reached 55.4 mg/L at the inoculation ratio of 5:1, which is 248% higher than the BTP1:BD co-culture without the biosensor-assisted cell selection system. The use of the biosensors in the mono-culture controls showed a different effect on the indigo production. The indigo concentrations of the engineered mono-culture strains BMS and BMS1 were 10.6 mg/L and 5.9 mg/L, respectively, both of which are lower than the BMC strain without any cell selection system. This result implied that the biosensor-assisted cell selection system may not be helpful for the biosynthesis in monocultures, which is consistent with the previous findings [16, 18].

**Fig. 4:**
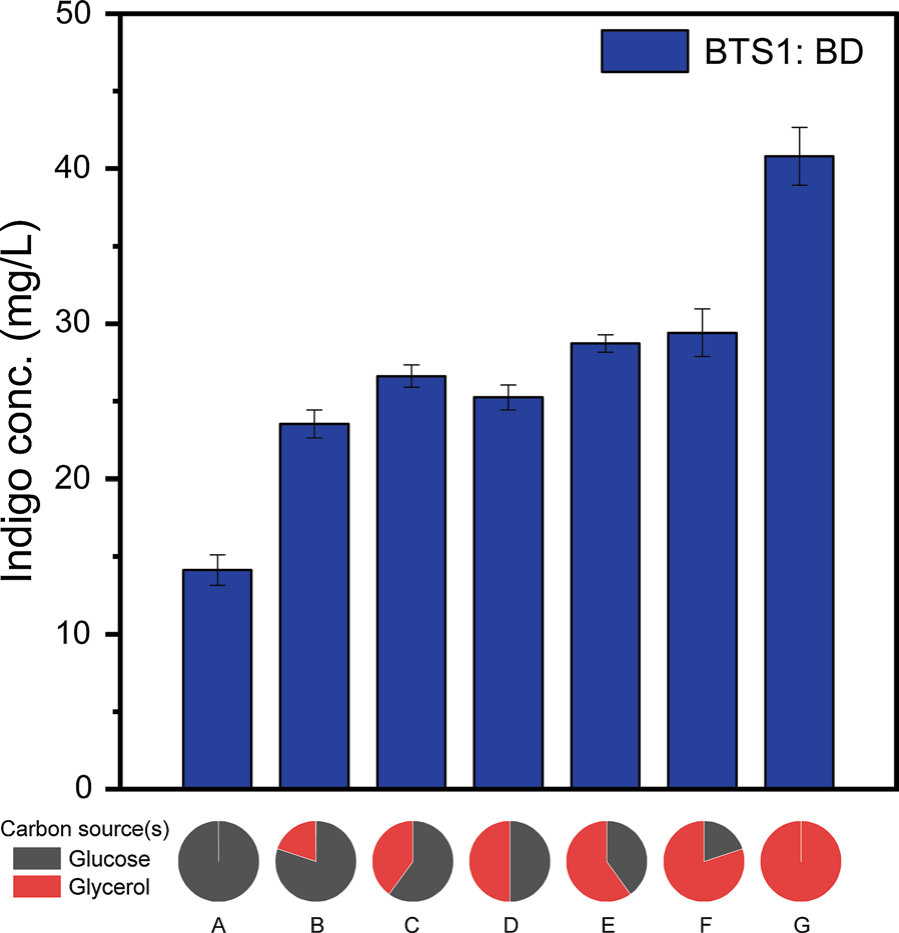
Comparison of the indigo bioproduction by the BTS1:BD co-culture grown on (A) 5 g/L glucose, (B) 4 g /L glucose and 1 g/L glycerol, (C) 3 g/L glucose and 2 g/L glycerol, (D) 2.5 g/L glucose and 2.5 g/L glycerol, (E) 2 g/L glucose and 3 g/L glycerol, (F) 1 g/L glucose and 4 g/L glycerol, and (G) 5 g/L glycerol. The co-culture strains were inoculated at the 1:1 ratio. The error bars represent the standard error of at least three biological replicates

Furthermore, we investigated the biosynthesis performance of the BTS1:BD co-culture in a series of medium containing different glucose/glycerol ratios. As shown in Fig. 4, when glucose was the sole carbon source, the indigo production was only 14.1 mg/L. The increase of the glycerol content in the substrate gradually improved the production. The indigo concentration was increased to 40.8 mg/L when glycerol was the sole carbon source, which is 190% higher than the production on glucose only. The results hereby are consistent with the previous section and indicate that glycerol is a preferred carbon substrate for the indigo biosynthesis. As such, glycerol was adopted for the following experiments.

### Using global transcription machinery engineering (gTME) to strengthen the co-culture’s biosynthesis performance

**Fig. 5:**
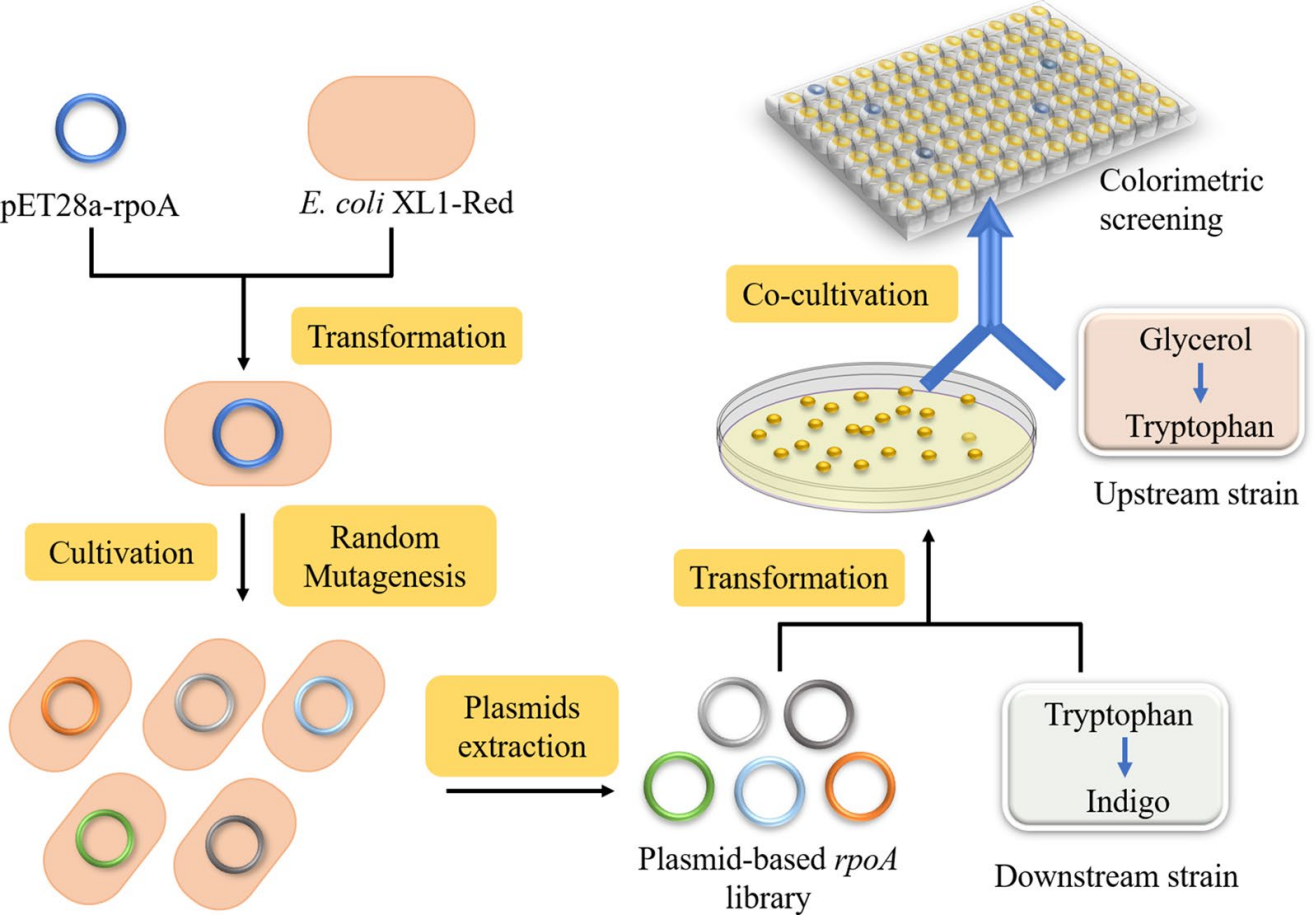
The Schematic of the screening strategy for identifying high-performance co-culture systems. *E. coli* XL1-Red was used to generate the *rpoA* mutant library, and a colorimetric assay was used for high throughput screening

Next, we sought to promote the biosynthetic capability of the downstream strain in the context of the co-culture. To this end, we adopted a novel approach based on global transcription machinery engineering (gTME) to alter the cellular transcriptome and introduce phenotypic diversity. A facile colorimetric assay was then utilized to identify the mutants with desired biosynthesis enhancement. The schematic design of this experiment is shown in Fig. 5. Specifically, *E. coli* gene *rpoA* encoding the α subunit of the RNA polymerase was randomly mutated through replication in *E. coli* XL1-Red which is capable of in vivo DNA mutagenesis. A *rpoA* mutants’ library was thus created and introduced into the downstream strain, generating 409 variants. Each of these downstream strain variants expressed a particular *rpoA* mutant and was then co-cultured with the upstream strain BTR1 at 1:1 inoculation ratio in 96-well plates. A colorimetric assay was used to rapidly screen the co-cultures to identify the ones with improved indigo production. Notably, since indigo has a distinctive blue color, high-performing co-culture variants can be distinguished by monitoring the blue color intensity in a high throughput manner.

**Fig. 6:**
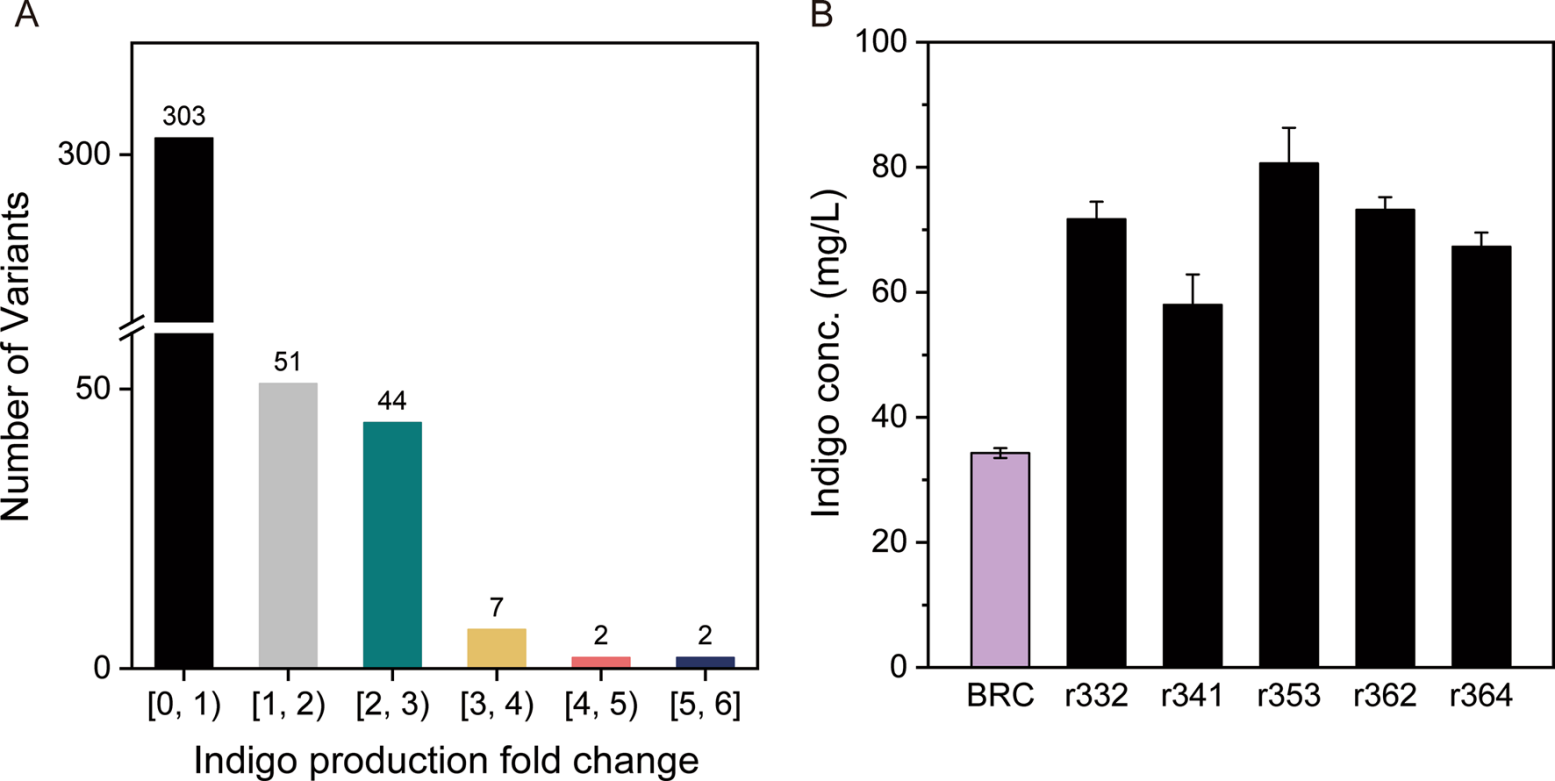
The indigo bioproduction by the co-cultures containing the *rpoA* mutants. **A** The statistical analysis of the biosynthesis performance difference between the co-culture variants grown in 96-well plate. **B** Comparison of the indigo production by the top five co-culture variants. The upstream and downstream strains were inoculated at 1:1 ratio. The error bars represent the standard error of at least three biological replicates

The statistical analysis of the biosynthesis performance difference between the co-culture variants grown in 96-well plate was shown in Fig. 6A. Most variants (303 of 409) presented a lower productivity compared to the control group, 106 variants exhibited improvement in the indigo bioproduction (which is about 25% of total mutations), indicating that this mutation strategy is efficient. 9 variants showed significant improvement with the indigo production fold change higher than 3. Top five variants (the indigo concentration greater than 12 mg/L in 96-wells plate) were chosen to verify their capability by co-cultured with the upstream strain BTR1 in the culture tubes. As shown in Fig. 6B, all top five mutants showed higher production performance than the control co-culture BTR1:BRC expressing the wild-type *rpoA*. The best co-culture variant, BTR1: BRM-353 (harboring the *rpoA*^353^mutant), produced 80.6 mg/L indigo from 5 g/L glycerol after 48 h cultivation, which was 135% higher than the BTR1:BRC control co-culture. These findings suggested that desirable phenotypes for high indigo biosynthesis in the co-culture were successfully created using gTME. To our knowledge, this is the first report utilizing gTME in the context of microbial co-cultures, offering a new research avenue to advance microbial co-culture engineering.

### Characterization of the identified downstream strain mutant

To characterize the *rpoA*^353^ mutant, its DNA sequence was analyzed. It was found that there are more than 700-point mutations in both the promoter and coding region of the *rpoA*^353^ mutant, which accounts for more than 50% of the original *rpoA* gene. This clearly indicates that the DNA mutagenesis method using the XL1-Red strain is highly efficient. Since *rpoA* is a key component for the global transcription, such a high degree of the mutation is considered to create strong perturbations for the cellular behaviors.

**Fig. 7:**
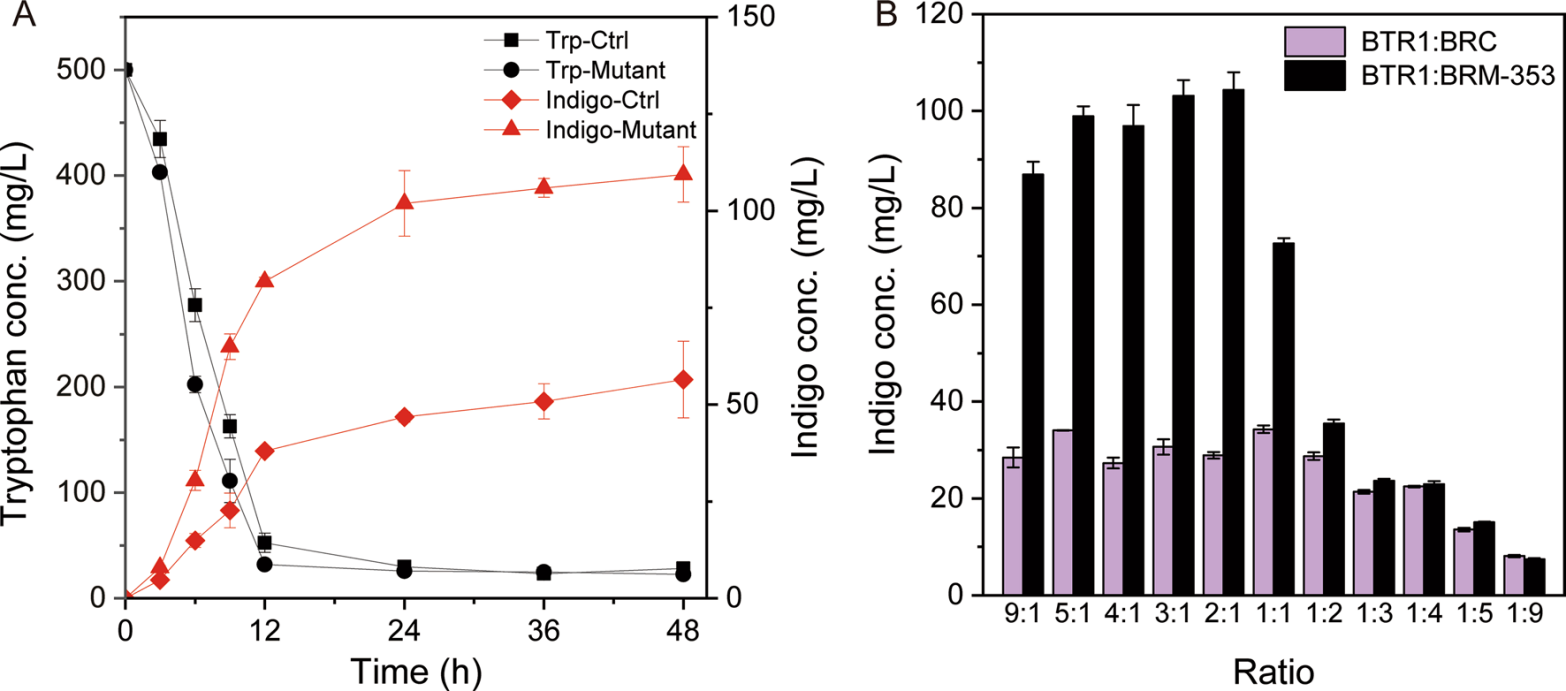
Characterization of BRM-353 mutant’s biosynthesis capability. **A** Time profiles of the tryptophan consumption and indigo production by BRC (control) and BRM-353 (mutant) fed with exogenous tryptophan. **B** The effect of the inoculation ratio on the indigo production by BTR1:BRC and BTR1:BRM-353 co-cultures. The error bars represent the standard error of at least three biological replicates

Strain BRM-353 harboring the *rpoA*^*353*^ mutant was characterized for its growth and tryptophan bioconversion capability. Specifically, BRM-353 and BRC (control strain harboring the wild-type *rpoA* gene) were both fed with 500 mg/L exogenous tryptophan and cultivated as mono-cultures at 30 °C. It was found that the mutant strain did not display any significant difference in the growth pattern compared with the control strain BRC (Fig. 7A). Moreover, the dynamic changes of tryptophan and indigo concentrations over time were monitored for 48 h. As shown in Fig. 7A, tryptophan in both BRC and BRM-353 cultures was quickly depleted after 12 h, and their consumption rates followed a similar pattern. Interestingly, these two strains showed different indigo production behavior. The indigo concentration in the BRM-353 culture increased rapidly 3 h after the inoculation and plateaued at around 101 mg/L after 24 h, whereas the control strain’s indigo biosynthesis peaked at around 50 mg/L. The results hereby confirm that the mutant strain BRM-353 possesses strong capability of producing indigo from tryptophan.

Next, the indigo biosynthesis by the identified BTR1:BRM-353 co-culture was optimized by varying the inoculation ratio to coordinate the biosynthetic strengths of the upstream and downstream pathway modules. As shown in Fig. 7B, for most of the tested inoculation ratios, the indigo production was significantly higher than the control co-culture BTR1:BRC using the wild type *rpoA*. The highest indigo concentration of 104.3 mg/L was achieved at the inoculation ratio of 2:1, which is 204% higher than the optimum of the BTR1:BRC co-culture and 1142% higher than the starting indigo-producing strain. The result confirmed the effectiveness of gTME in co-culture engineering. More importantly, it reveals that, even when the biosynthetic pathway is not further modified, the metabolic status of a co-culture strain can be altered by engineering strategies such as gTME to trigger the biosynthesis enhancement.

We also compared the growth profiles of the co-cultures before and after the introduction of the *rpoA*^*353*^ mutant. The strain-to-strain ratio within both co-cultures’ populations (Additional file 1: Fig. S3) only fluctuated to a small degree throughout the cultivation, indicating the stability of the co-culture system. There are no significant differences between the two co-cultures’ growth profiles, which suggests that gTME did not change the interaction between the co-culture strains for their co-growth behavior.

## Discussion

Glucose is a commonly used carbon source for microbial biosynthesis [30, 31], while our study shows that glycerol is a preferred carbon source for the indigo biosynthesis. It has been found that the expression and activity of TnaA, a key pathway enzyme, is inhibited by glucose [32]. As a result, the use of glucose limited the formation of the pathway intermediate indole, leading to the low indigo production on glucose. The use of glycerol avoided such inhibition on TnaA and caused higher tryptophan conversion and indigo production.

On the other hand, the integration of the biosensor-assisted cell selection system into the upstream strain helped eliminate the low-performing cells of the upstream strain’s subpopulation and thus improved the tryptophan provision for the indigo production. The two tested biosensor systems showed comparable performance for the indigo biosynthesis improvement. However, the use of TrpR-P*mtr*-*hipA* system did not require the addition of any exogenous antibiotic for cell selection, which is considered advantageous for process economy, especially for applications in larger scale process. Notably, this strategy requires TrpR (encoded by *trpR* on the chromosome) as the sensor protein. The *tnaC-tetA* system, however, does not need additional sensor proteins for the sensing mechanism to work. Both systems have their advantages and have been utilized for facilitating microbial biosynthesis in previous studies [16, 21].

gTME has been demonstrated to be an effective technique for introducing diverse changes into the global metabolic network [20, 33]. Previous gTME studies have been mainly pursued using the mono-culture of a single microbial strain. In this work, we implemented gTME within the context of a microbial co-culture, which expands the application of this powerful approach. Different from the conventional approach using error-prone PCR to carry out random DNA mutagenesis, this study recruited *E. coli* XL1-RED with hyper mutation efficiency for generating *rpoA* gene mutants in vivo. The resulting *rpoA* mutant genes, once introduced into the downstream strain, changed its global transcriptome and diversified the phenotypes, some of which are helpful for the indigo production in the context of the co-culture. For the best mutant *rpoA*^*353*^, 50% of the gene sequence is different from the wild type *rpoA*. As such, it is highly challenging to track which specific mutations caused the phenotypic changes. Notably, the characterization of the identified mutant showed that its biosynthesis improvement can be attributed to the enhanced tryptophan-to-indigo conversion capability (Fig. 7A) and its growth behavior was still similar to the wild type strain (Additional file 1: Fig. S4). It is postulated that such enhancement is because the *rpoA* mutation downregulated of the competing pathway(s) in the background metabolism or altered cellular environment that better supports the pathway enzymes’ in vivo activities. However, how exactly the transcriptomic changes induced by *rpoA*^*353*^ contribute to such enhancement remains unknown and needs to be further investigated.

Traditional mutant library screening methods are also based on the use of mono-cultures. For example, a library of mutant strains is fed with exogenous metabolite precursors for identification of individual mutants with high conversion. However, it is possible that the identified mutants may not co-grow well with the upstream strain in the co-culture setting, resulting in negative screening results. For this study, the screening of the mutant library was conducted using the co-cultures, i.e. by co-cultivating the downstream strain mutants with the upstream strain. Such a screening method considers the metabolic interaction between the co-culture strains for completing the indigo biosynthesis, which eliminated the issue associated with mono-culture-based approach and thus improved the screening process efficiency. To this end, the results of this work lay a foundation for using similar strategies for engineering other microbial co-cultures to improve biosynthesis performance in future studies.

## Conclusion

In this work, we combined several metabolic engineering strategies to establish and improve the indigo biosynthesis in *E. coli*. Glycerol was found to be a better carbon substrate than glucose, and employment of modular co-culture engineering strategies resulted in superior production performance over the mono-culture approach. On top of these efforts, biosensor-assisted cell selection was introduced to the upstream co-culture strain to strengthen the tryptophan provision, whereas global transcription machinery engineering was adopted to enhance the downstream strain’s capability for converting tryptophan to indigo. When integrated together, these strategies led to the production of 104.3 mg/L indigo, 11.4-fold higher than that of the starting *E. coli* strain.

## Materials and methods

### Plasmids and strains construction

Plasmids and strains used in this work are listed in Tables 1 and 2. Flavin-containing monooxygenase (FMO) from *Methylophaga aminisulfidivorans* was codon-optimized and synthesized by Bio basic Inc. Restriction enzymes, DNA ligase, and DNA polymerase were purchased from New England Biolabs. Plasmid extraction kit and DNA purification kit were purchased from Zymo Research. Plasmid construction was conducted using DH5α competent cells (New England Biolabs), and mutation was performed by using XL1-Red cells (Agilent). Sequences of the primers used in this study are listed in Additional file 1: Table S1.

**Table1:** Plasmids used in this study

Plasmid	Description	Source
pET28a	T7 promoter, Kan^R^	Novagen
pACYCDuet-1	Double T7 promoters, Cm^R^	Novagen
pET21c	T7 promoter, Amp^R^	Novagen
pCDFDuet-1	T7 promoter, Strp^R^	Novagen
pTE3	pET28a carrying the *trpE*^*fbr*^ and *aroG*^*fbr*^ genes under the control of the constitutive *Zymomonas mobilis* pyruvate decarboxylase promoter (P*pdc*)	[21]
pTD2	pACYC Duet carrying the *trpD*, *trpC*, *trpB* and *trpA* genes under the control of the constitutive *Zymomonas mobilis* pyruvate decarboxylase promoter (P*pdc*)	[21]
pSE	pET21c carrying tryptophan regulatory element (*tnaC* operon) and the *tetA* gene, Amp^R^	[16]
pSE1	pET21c carrying the *hipA* gene under the control of the *mtr* promoter (P*mtr*)	[21]
pDF	pET28a carrying the *fmo* gene (from *Methylophaga aminisulfidivorans*) under control of T7 promoter	This study
pMF	pCDFDuet-1 carrying the *fmo* gene (from *Methylophaga aminisulfidivorans*) under control of T7 promoter	This study
pDT	pET21c carrying the t*etA* gene under the control of the *tetA* promoter (P*tet*)	This study
pTS0	pET28a carrying the *proA* promoter with the *kanR* gene replaced by the *strpR* gene	This study
pTS1	pTS0 carrying *rpoA* gene under control of *proA* promoter	This study
pTS2	pTS1 after random mutagenesis	This study

**Table 2:** Strains used in this study

Strain	Description	Source
BH2	*E. coli* BL21(DE3) *ΔxylA ΔtyrA ΔpheA*	[22]
XL1-Red	F-*endA*1 *gyrA*96(nal^R^) *thi*-1 *relA*1 *lac glnV*44 *hsdR*17(r_K_^−^ m_K_^+^) *mutS mutT mutD*5 Tn10	Agilent
BTP1	BH2 harboring pTE3, pTD2 and pET21c	[21]
BTS	BH2 harboring pTE3, pTD2 and pSE	[16]
BTS1	BH2 harboring pTE3, pTD2 and pSE1	[21]
BTR1	BTS1 harboring pTS	This study
BF	BH2 harboring pDF	This study
BD	BH2 harboring pACYCDuet-1, pDT and pDF	This study
BRC	BD harboring pTS1	This study
BRM	BD harboring pTS2	This study
BRM-353	BD harboring pTS2with the *rpoA*^*353*^ mutant	This study
BMC	BTP1 harboring pMF	This study
BMS	BTS harboring pMF	This study
BMS1	BTS1 harboring pMF	This study
XLR	XL1-Red harboring pTS1	This study

To construct plasmid pDF, the *fmo* gene from *Methylophaga aminisulfidivorans* was codon-optimized and synthesized by Bio Basic Inc. The *fmo* gene was digested by NdeI and XhoI then ligated with pET28a treated with the same enzymes. To construct plasmid pMF, the *fmo* gene was digested by NdeI and XhoI then ligated with pCDFDuet-1 treated with the same enzymes.

For the construction of plasmid pDT, the DNA fragment containing promoter *Ptet* and tetracycline resistance gene *tetA* was PCR amplified from plasmid pBR322 using primers tet-F and tet-R, then inserted into plasmid pET21c using the EcoRI and XhoI sites.

To construct plasmid pTS0, the DNA fragment containing promoter *Pstrp* and streptomycin resistance gene *strpR* was PCR amplified from plasmid pCDFDuet-1 using primers strp-F and strp-R. Using the EcoNI site, the PCR product was inserted into plasmid pET28a-*PproA* containing a weak constitutive proA promoter [34].

For the construction of plasmid pTS1, the *rpoA* gene was PCR amplified from the genome of *E. coli* K12 chromosome by primers rpoA-F and rpoA-R. The *rpoA* gene was digested by SpeI and XhoI then ligated with pTS0 treated with the same enzymes to generate pTS1.

To construct the mutation library of pTS2, pTS1 was transformed into XL1-Red for ten cultivation generations, the mutation pTS2 library was generated by plasmid extraction of the 10th cultivation.

### Medium and cultivation

For the production of indigo, strains were cultured in M9 special medium. One liter of M9 special medium contained 5 g glucose or glycerol, 0.5 g yeast extract, 8.5 g Na_2_HPO_4_•2H_2_O, 3 g KH_2_PO_4_, 1 g NH_4_Cl, 0.5 g NaCl, 40 mg tyrosine, 40 mg phenylalanine and trace elements. Trace elements included 0.4 mg/L Na_2_EDTA, 0.03 mg/L H_3_BO_3_, 1 mg/L thiamine, 0.94 mg/L ZnCl_2_, 0.5 mg/L CoCl_2_, 0.38 mg/L CuCl_2_, 1.6 mg/L MnCl_2_, 3.77 mg/L CaCl_2_, and 3.6 mg/L FeCl_2_.

All strains were pre-cultured overnight in LB medium with proper antibiotics at 37 °C, 250 rpm. For the mono-culture experiment, 2% (v/v) overnight LB cultures of the desired *E. coli* strains were inoculated in fresh LB medium with proper antibiotics for 6 h. Then cells were collected and re-suspended in M9 special medium containing appropriate antibiotics and 0.1 mM IPTG with an initial OD_600_ of 0.6. After cultivation at 30 °C for 48 h, samples were collected for analysis. For the co-culture experiment, 2% (v/v) overnight LB cultures of the desired upstream and downstream strains were inoculated in fresh LB medium for 6 h, respectively. Both upstream and downstream strains were collected and re-suspended in M9 special medium containing appropriate antibiotics and 0.1 mM IPTG. For the culture tube scale co-culture, the upstream and downstream strains were inoculated into 2 mL fresh M9 special medium with a series of desired ratios to reach an initial total OD_600_ of 0.6. For the 96 well plate scale co-culture, the upstream and downstream strains were inoculated into 200 μL M9 special medium in each well to reach an initial total OD_600_ of 0.6. The plates were sealed by aluminum sealing film (Platemax, Axygen). After cultivation at 30 °C and 250 rpm for 48 h, samples were collected for analysis.

For characterization of the downstream strain mutant BRM-353, both control strain and mutant strain were cultivated in M9 special medium (containing 5 g/L of glycerol) with additional 500 mg/L tryptophan. The OD_600_ value, the tryptophan concentration, and the indigo concentration at desired time points were measured.

To distinguish the upstream and downstream strains in the co-culture, the co-culture samples were collected, diluted with sterile water, and spread onto a set of LB agar plates containing kanamycin, streptomycin, and chloramphenicol. After overnight incubation, the colonies on the plates were transferred to a new set of LB agar plates containing tetracycline. Since the downstream strain is resistant to tetracycline and the upstream strain is not, the colonies that could grow on both sets of plates were counted as the downstream strains. The ratio between the colony numbers grown on the two sets of plates represents the ratio of the co-culture strains’ populations in the culture.

### Mutation of the *rpoA* gene

The plasmid pTS1 containing the wild-type *rpoA* gene was transformed into *E. coli* XL1-Red, resulting strain XLR. XLR was cultured in LB medium with 50 mg/L streptomycin at 37 °C, 250 rpm for 12 h, and the resulting culture was transferred into fresh LB medium with streptomycin (2% v/v). After ten generations of cultivation, a library of plasmid pTS1 mutants was generated and transformed into strain BD, yielding strain BRM. All BRM colonies were selected and characterized for the indigo biosynthesis by co-cultivation with the upstream strain BTR1.

### Extraction of indigo

The cell cultures were centrifuged at 5000 rpm for 10 min to collect the cell pellet. The precipitate was a mixture of cells, cell-bound indigo, and free indigo particles. After re-suspended in DMSO, mixture was sonicated on ice for 30 cycles; each cycle comprised 1-s sonication and 6-s break. The sonicated mixture was then centrifuged at 10,000 rpm for 5 min, and supernatant was collected for indigo analysis. The extraction process was repeated until the supernatant is free of blue color.

### Metabolite analysis

Pathway metabolites analysis were performed by high performance liquid chromatography using a Shimadzu Prominence HPLC System equipped with UV detector SPD-20AV. For tryptophan quantification, 1 mL of cell culture was centrifuged at 10,000 rpm for 5 min, and the supernatant was filtered through 0.45 µm PVDF membrane syringe filters (Whatman Inc). 10 μL of filtered sample was injected into a ZORBAX Eclipase Plus C18 column (4.6 × 150 mm, 5 μm, Agilent Technologies) and eluted by solvent A (0.5% acetic acid) and solvent B (100% acetonitrile). The total flow rate was maintained at 0.6 mL/min, and the following gradient was used for tryptophan separation: 0–10 min, a linear gradient of B from 0 to 50%; 10–10.2 min, a linear gradient of B from 50 to 100%; 10.2–13 min, 100% B; 13.1 min, a linear gradient of B from 100 to 0%; 13.1–16 min, 0% B (all in vol%). Absorption at 280_ nm_ was monitored for the tryptophan quantification.

For the indigo quantification, 1 mL extraction mixture was centrifuged at 10,000 rpm for 5 min, and the supernatant was filtered through 0.45 µm PVDF membrane syringe filters. 10 μL of filtered sample was injected into a Hamilton PRP-C18 column (4.6 × 150 mm, 5 mm, Hamilton, USA) and eluted by solvent A (0.5% acetic acid) and solvent B (100% acetonitrile). The total flow rate was maintained at 0.6 mL/min, and the following gradient was used: 0–5 min, a linear gradient of B from 60 to 100%; 5–20 min, a linear gradient of B from 100 to 60% (all in vol%). Absorption at 620_ nm_ was monitored for the indigo quantification.

## Supplementary Information

**Additional file 1. Table S1**. Sequences of the primers used in this study.** Fig. S1**. Comparison of indigo production at different temperatures. Strain BD containing the* fmo* gene was fed with 100mg/L of tryptophan or indole for the bioproduction. The error bars represent the standard error of at least three biological replicates.** Fig. S2**. Schematic illustration of cell selection using (A) TrpR-P*mtr-hipA* and (B)* tnaC-tetA* systems.** Fig. S3**. The strain-to-strain ratio change over time within the populations of (A) the BTR1:BRC co-culture and (B) BTR1:BRM-353 co-culture.** Fig. S4**. Growth curves of the downstream strains BRC (control) and BRM-353 (mutation). Both strains were cultured in shake flask with M9 medium containing 5 g/L glycero

## Data Availability

The datasets used and/or analyzed during the current study are available from the corresponding author on reasonable request.
