# Supplementary material for: Development and optimization of a microbial co-culture system for heterologous indigo biosynthesis
Source: Microb Cell Fact. 2021 Aug 4;20:154. doi: 10.1186/s12934-021-01636-w (PMC8336371; doi:10.1186/s12934-021-01636-w)
Supplement: Supplementary file 1 — Additional file 1. Table S1. Sequences of the primers used in this study. Fig. S1. Comparison of indigo production at different temperatures. Strain BD containing the fmo gene was fed with 100mg/L of tryptophan or indole for the bioproduction. The error bars represent the standard error of at least three biological replicates. Fig. S2. Schematic illustration of cell selection using (A) TrpR-Pmtr-hipA and (B) tnaC-tetA systems. Fig. S3. The strain-to-strain ratio change over time within the populations of (A) the BTR1:BRC co-culture and (B) BTR1:BRM-353 co-culture. Fig. S4. Growth curves of the downstream strains BRC (control) and BRM-353 (mutation). Both strains were cultured in shake flask with M9 medium containing 5 g/L glycero [file 12934_2021_1636_MOESM1_ESM.docx]

Additional file for

**Development and optimization of a microbial co-culture system for heterologous indigo biosynthesis**

Table S1. Sequences of the primers used in this study

(Underlined sequences indicate restriction sites.)

| Primer name | Primer Sequence  (5’-3’) |
| --- | --- |
| tet-F | AGCATATGAACAAATAGGGGTTCCGC |
| tet-R | AGCTCGAGTTCCATTCAGGTCGAGGT |
| strp-F | AGCCTGAATCAGGTCATCGTGGCCGGATCTT |
| strp-R | AGCCTGATTCAGGGAGTGAGCTAGCTATTTG |
| rpoA -F | AGACTAGTATGCAGGGTTCTGTGACA |
| rpoA -R | AGCTCGAGTTACTCGTCAGCGATGCT |


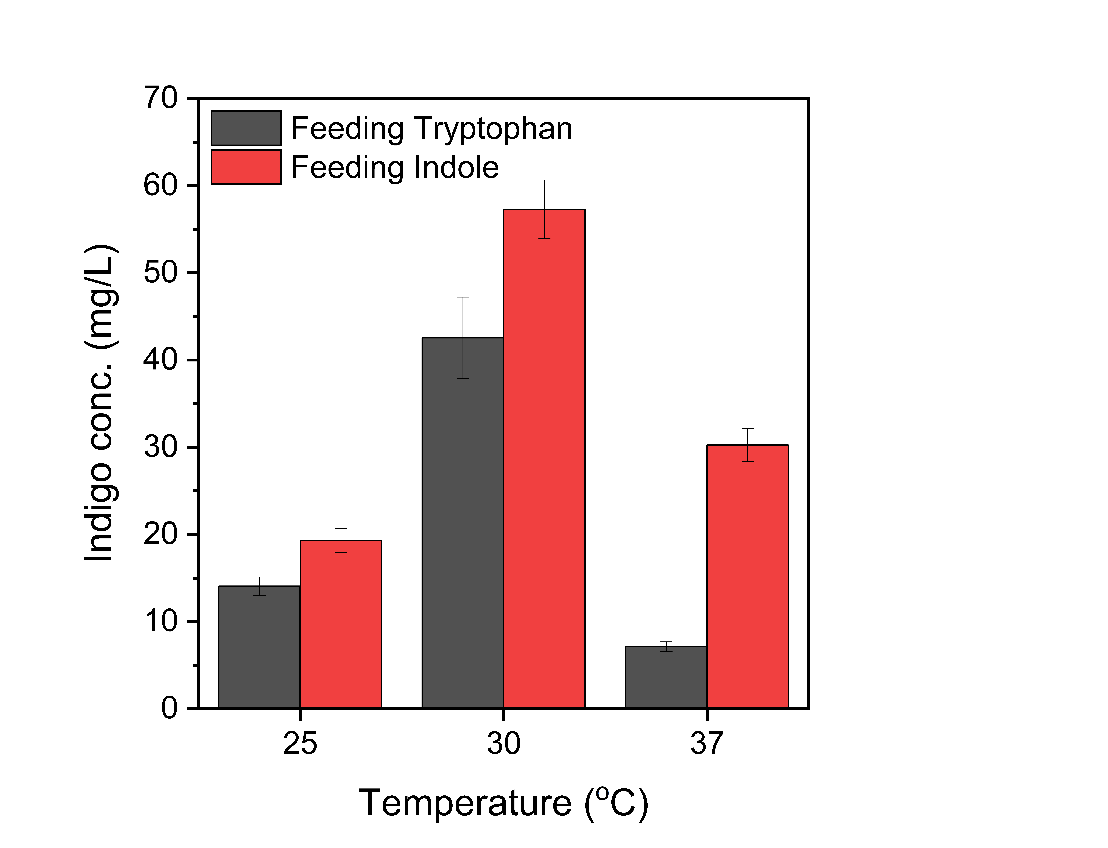


Fig. S1 Comparison of indigo production at different temperatures. Strain BD containing the *fmo* gene was fed with 100mg/L of tryptophan or indole for the bioproduction. The error bars represent the standard error of at least three biological replicates.


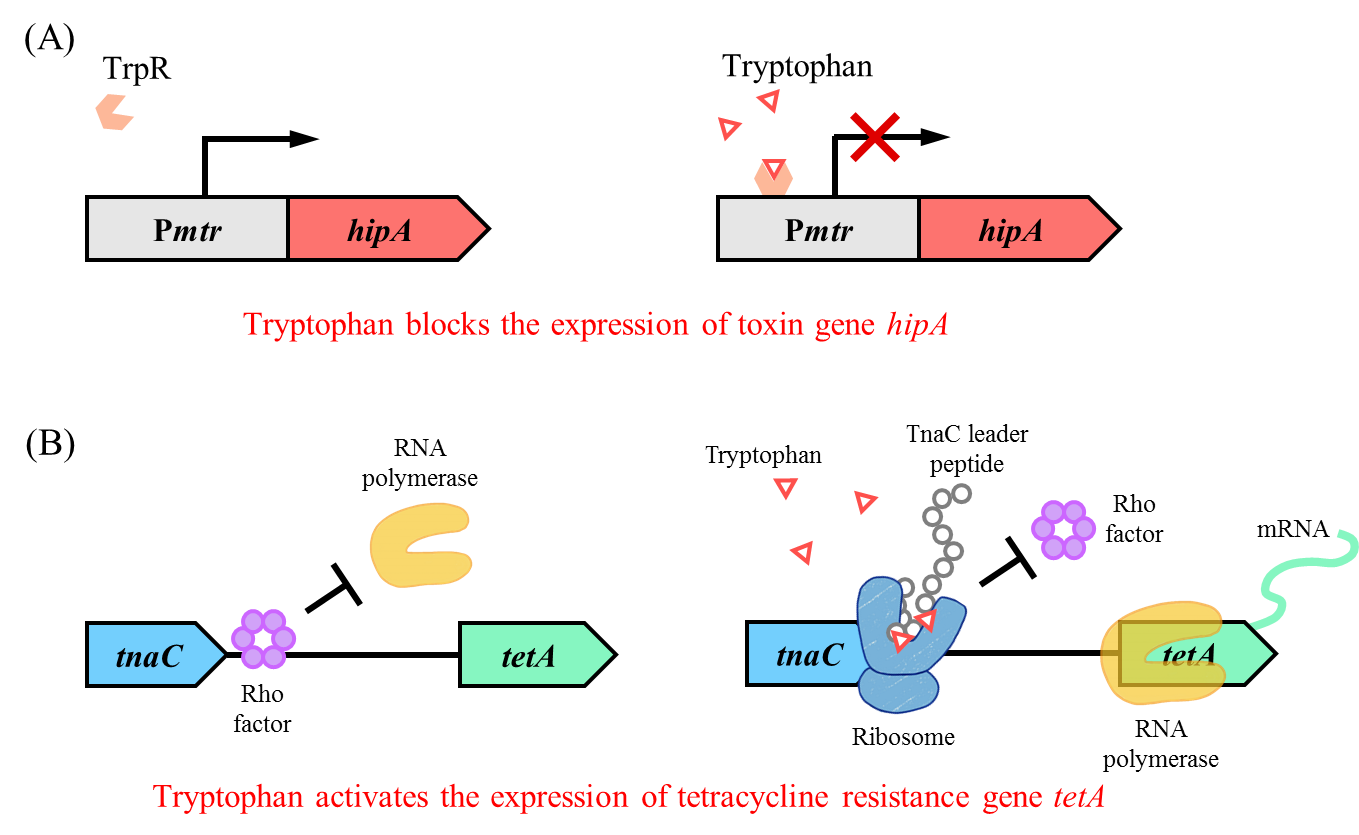


Fig. S2 Schematic illustration of cell selection using (A) TrpR-P*mtr*-*hipA* and (B) *tnaC*-*tetA* systems.

| (A) | (B) |
| --- | --- |
| 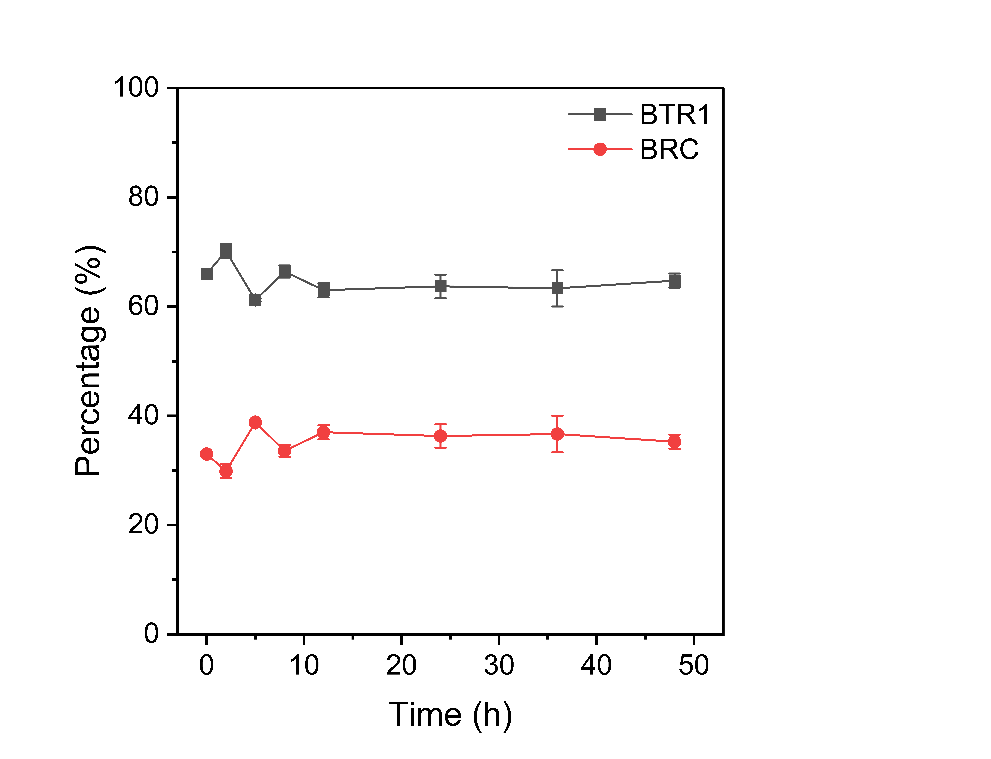 | 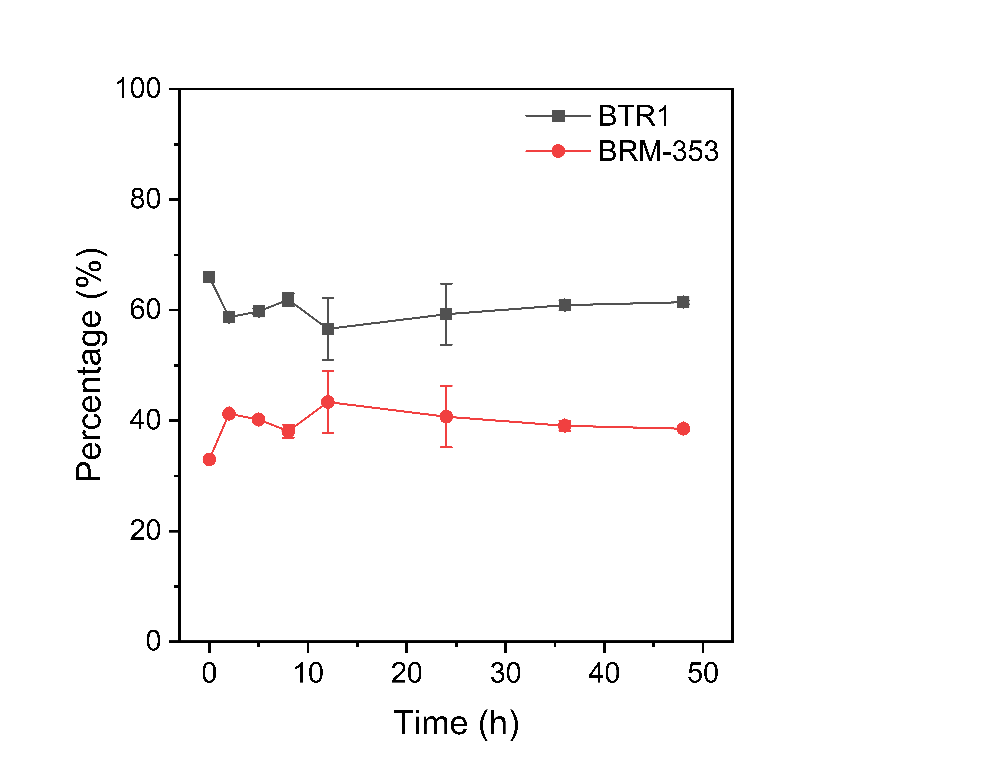 |

Fig. S3 The strain-to-strain ratio change over time within the populations of (A) the BTR1:BRC co-culture and (B) BTR1:BRM-353 co-culture.

Fig. S4 Growth curves of the downstream strains BRC (control) and BRM-353 (mutation). Both strains were cultured in shake flask with M9 medium containing 5 g/L glycerol.
